# An automated pheochromocytoma and paraganglioma lesion segmentation AI-model at whole-body ^68^Ga- DOTATATE PET/CT

**DOI:** 10.1186/s13550-024-01168-5

**Published:** 2024-11-05

**Authors:** Fahmida Haque, Jorge A. Carrasquillo, Evrim B. Turkbey, Esther Mena, Liza Lindenberg, Philip C. Eclarinal, Naris Nilubol, Peter L. Choyke, Charalampos S. Floudas, Frank I. Lin, Baris Turkbey, Stephanie A. Harmon

**Affiliations:** 1 Artificial Intelligence Resource, National Cancer Institute, National Institutes of Health, Bethesda, MD 20814 USA; 2 Molecular Imaging Branch, National Cancer Institute, National Institutes of Health, Bethesda, MD 20814 USA; 3 Radiology and Imaging Sciences, Clinical Center, National Institutes of Health, Bethesda, MD 20892 USA; 4 Surgical Oncology Program, National Cancer Institute, National Institutes of Health, Bethesda, MD 20892 USA; 5 Center for Immuno-Oncology, National Cancer Institute, National Institutes of Health, Bethesda, MD 20892 USA

**Keywords:** DOTATATE PET/CT, Lesion segmentation, Machine learning, Image processing, Artificial intelligence, Deep learning, Oncology, Neuroendocrine, Pheochromocytoma and paraganglioma, PPGL

## Abstract

**Background:**

Somatostatin receptor (SSR) targeting radiotracer ^68^Ga-DOTATATE is used for Positron Emission Tomography (PET)/Computed Tomography (CT) imaging to assess patients with Pheochromocytoma and paraganglioma (PPGL), rare types of Neuroendocrine tumor (NET) which can metastasize thereby becoming difficult to quantify. The goal of this study is to develop an artificial intelligence (AI) model for automated lesion segmentation on whole-body 3D DOTATATE-PET/CT and to automate the tumor burden calculation. 132 ^68^Ga-DOTATATE PET/CT scans from 38 patients with metastatic and inoperable PPGL, were split into 70, and 62 scans, from 20, and 18 patients for training, and test sets, respectively. The training set was further divided into patient-stratified 5 folds for cross-validation. 3D-full resolution nnUNet configuration was trained with 5-fold cross-validation. The model’s detection performance was evaluated at both scan and lesion levels for the PPGL test set and two other clinical cohorts with NET (*n* = 9) and olfactory neuroblastoma (ONB, *n* = 5). Additionally, quantitative statistical analysis of PET parameters including SUVmax, total lesion uptake (TLU), and total tumor volume (TTV), was conducted.

**Results:**

The nnUNet AI model achieved an average 5-fold validation dice similarity coefficient of 0.84 at the scan level. The model achieved dice similarity coefficients (DSC) of 0.88, 0.6, and 0.67 at the scan level, the sensitivity of 86%, 61.13%, and 61.64%, and a positive predictive value of 89%, 74%, and 86.54% at the lesion level for the PPGL test, NET and ONB cohorts, respectively. For PPGL cohorts, smaller lesions with low uptake were missed by the AI model (*p* < 0.001). Anatomical region-based failure analysis showed most of the false negative and false positive lesions within the liver for all the cohorts, mainly due to the high physiologic liver background activity and image noise on ^68^Ga- DOTATATE PET scans.

**Conclusions:**

The developed deep learning-based AI model showed reliable performance for automated segmentation of metastatic PPGL lesions on whole-body ^68^Ga-DOTATATE-PET/CT images, which may be beneficial for tumor burden estimation for objective evaluation during therapy follow-up.

https://www.clinicaltrials.gov/study/NCT03206060, https://www.clinicaltrials.gov/study/NCT04086485, https://www.clinicaltrials.gov/study/NCT05012098.

**Supplementary Information:**

The online version contains supplementary material available at 10.1186/s13550-024-01168-5.

## Introduction

Pheochromocytomas/paragangliomas (PPGL), are rare neuroendocrine tumors (NETs) arising from the adrenal medulla or extraadrenal chromaffin tissue [1]. These tumors produce catecholamines which can produce severe, prolonged hypertension resulting in life-threatening complications such as end organ damage, severe acute coronary syndromes, and stroke [2, 3]. 71% of the metastatic PPGL patients develop bone metastases, 81% of them exclusively in the spine [4], which destroy the skeletal tissue causing acute and chronic skeletal-related events (SREs), can be painful and reduce quality of life. Within a mean of 4.3 months of diagnosis of bone involvement in metastatic PPGL patients, the first SREs can develop along with further spine injuries with progression [4]. Metastasized PPGLs can be surgically inoperable and resistant to treatment if diagnosed late. Therefore, early intervention and disease identification are critical to mitigate the complications and deploy proper treatment plans.

PPGL tumors commonly exhibit overexpression of somatostatin receptors (SSTR subtype 2) similar to other NET tumors. Exploiting the presence of SSTR2 on tumor cells, targeted radionuclide-somatostatin analogs (SSAs) are being used for molecular imaging and peptide receptor radionuclide therapy (PRRT) of PPGLs [5]. ^68^Ga-DOTA(0)-Tyr(3)-octreotate (^68^Ga-DOTATATE), an SSA, is predominantly used in integrated positron emission tomography/computed tomography (PET/CT) for the detection and administration of patients with NET [5]. A recent study showed that ^68^Ga-DOTATATE PET/CT had a sensitivity of 98.7% per lesion detection rate of spine bone metastases, outperforming other imaging modalities such as whole-body CT, whole-body magnetic resonance imaging (MRI), spine MRI, ^18^F-fluoro-2-deoxy-d-glucose (^18^F-FDG) PET/CT [2].

Traditionally, quantitative assessment of PET/CT uptake relies on manual or threshold-based assessment by nuclear medicine physicians and radiologists. This time-consuming process is burdensome but provides useful information for tracking lesion growth, total tumor burden, and monitoring treatment response in PPGLs, as the spread of the metastatic PPGLs can be aggressive resulting in a large number of tumors with varying sizes in a single scan [6–8]. Therefore, monitoring quantitative change in tumor burden, while important, is usually not feasible in a busy clinical workflow [8]. The medical imaging research community has recognized the importance of automating segmentation tools for both clinical management and research.

In 2015, Ronneberger et al. [9] introduced convolutional neural network-based architecture for medical image segmentation called U-Net which has driven the research in artificial intelligence (AI)-based segmentation models for medical images, including now-popular modifications such as nn-Unet [10]. These common architectures have shown success for lesion segmentation, prognosis prediction, organ segmentation, etc. in ^18^F-FDG PET/CT [11]. However, unlike FDG-PET/CT, fewer AI models have been developed for ^68^Ga-DOTATATE PET/CT in the literature. As the variety of PET agents increases, there is more need for segmentation tools adapted uniquely to specific agents. In this study, we aim to develop and evaluate an AI model for automated PPGL lesion segmentation using deep learning (DL) techniques on whole-body ^68^Ga-DOTATATE-PET/CT images.

## Materials and methods

### Study population

In this study, three independent clinical trial populations were used to develop and validate the AI model. Detailed information about the DOTATATE PET/CT scans of all three cohorts is listed in Table 1.

**Table 1:** Information on PET/CT images on different cohorts

	PPGL	NET	ONB
Number of patients	38	9	5
Total Number of Scans	126	11	8
Median Number of Scan per patient	3 [1, 9]	1 [1, 3]	2 [1, 2]
Median Number of lesions per scan	30 [4, 277]	18.5 [3, 68]	8 [3, 17]
Median total dose [MBq]	195.36 [119.14, 463.24]	170.47 [142.3, 206.8]	178.34 [143.93, 212.01]
Median Pixel Spacing [mm]	1.46 [1.46, 3.65]	2.73 [2.73, 3.18]	3.18
Median Slice Thickness [mm]	1.5 [1.5, 3.27]	3.27 [3, 3.27]	3
Gender [M/F]	22/14	5/6	4/1
Median Height [m]	1.74 [1.52, 1.94]	1.71 [1.51, 1.85]	1.77 [1.5, 1.9]
Median Age [years]	53 [16, 73]	46 [39, 66]	57 [51, 61]
Median Weight [kg]	81 [47, 189]	77.1 [53.60, 99]	89.9 [52.3 99.4]
Manufacturer	SIEMENS GE MEDICAL SYSTEMS	SIEMENS GE MEDICAL SYSTEMS	SIEMENS
Model Name (no of scans)	Biograph 64_Mct (32) petmct1(71) petmct2 (22) Discovery MI DR (1) Biograph128_mCT (2) Tippett (2) Biograph128_mCT 4R (1) Discovery MI (3) Biograph64_Vision 600 (1)	Discovery MI DR (9) Biograph128_mCT (2)	Biograph128_mCT (6) Biograph128_mCT 4R (2)

The first cohort consists of 38 adult patients undergoing Lu-177-DOTATATE treatment for inoperable, metastatic, and histologically proven Pheochromocytoma/Paraganglioma (NCT03206060), referred to as *PPGL cohort* for the remainder of the study. Patients underwent baseline imaging with ^68^Ga-DOTATATE, followed by additional imaging after two and four treatment administrations, respectively. This cohort has been utilized to develop the AI model.

The second cohort consists of 9 adult patients with inoperable Gastroenteropancreatic Neuroendocrine Tumors (GEP-NET) are referred to as *NET* patients (NCT04086485). The patients underwent Lu-177-DOTATATE (Lutathera) in combination with Olaparib treatment. All patients underwent baseline Ga[68]-DOTATATE PET/CT at 32 weeks after treatment administration and then every 24 weeks in the follow-up period. The third cohort consists of 5 patients with histologically confirmed recurrent or metastatic Olfactory neuroblastoma from a phase 2 clinical trial (NCT05012098) and are referred to as *ONB* patients. These patients underwent the immunotherapy drug Bintrafusp Alfa treatment. All patients underwent baseline Ga[68]-DOTATATE PET/CT imaging within 28 days prior to the first drug administration, and follow-up scans every 8 weeks (+/- 1 week).

### Expert annotations

Ground truth expert annotations were generated using a threshold-based Universal Lesion ID function in semi-automated commercial MIM software (Cleveland, OH, USA) which applies a global threshold to the image and then separates spatially distinct regions into unique lesions for the accept/reject procedure. Due to the high disease burden within all cohorts, nuclear medicine physicians utilized the software to generate an automated global threshold-based segmentation of any uptake areas with SUVmax ≥ 6 for PPGL and NET cohorts, and SUVmax ≥ 4 for the ONB cohort. Experts then reviewed all suggested regions and excluded any normal tissue uptake or indeterminate/non-disease-related uptake areas. Experts also added any tumor areas that might have been missed due to having SUVmax less than the threshold based on the radiology reports of the patients when available. Thus, the difference in thresholding approach in different cohorts should not have any implication on the contours. The final contours were considered as true positive tumors per the clinical protocol.

### Image processing

Before preparing the dataset for the AI model development, the following steps were taken to process the scans. Initially, all the DICOM data was converted into the Neuroimaging Informatics Technology Initiative (NIfTI) format using publicly available methods [12, 13]. The raw PET images were converted to standardized uptake values (SUV). CT image volumes and binary masks reflecting expert annotations were resampled to PET volume size and resolution. Segmentation masks were exported from MIM software in the DICOM RT-Struct file and the Python package, dcmrtstruct2nii [14] was used to convert the DICOM RT-Struct to the NIfTI format.

For AI development, the PPGL dataset was the only cohort used for training and all other cohorts were reserved for external validation. 132 scans from 38 PPGL patients, were split into 70, and 62 scans, from 20, and 18 patients for training, and test sets, respectively. The training utilized 5-fold cross-validation from the training set with 70 scans (56 scans from 15 patients for training and 14 scans from 5 patients for validation) from 20 patients. Patients with multiple scans were put into one of the sets (train, test, or validation) to make sure no patients were present in two sets. This was done to avoid overfitting the model and remove patient-level biases. The test set was reserved for model evaluation.

### AI model development

**Fig. 1:**
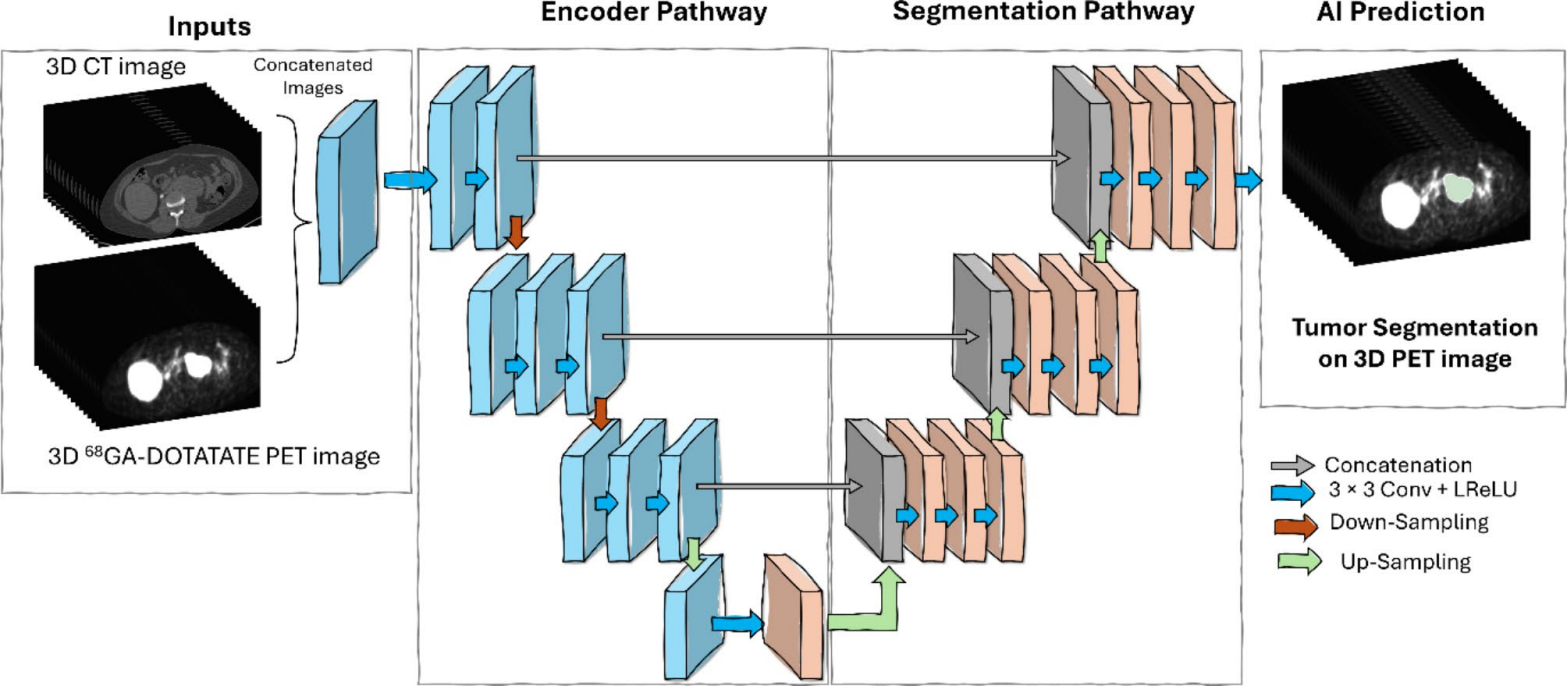
Workflow of 3D full resolution nnUNet model for automated 68GA-DOTATATE-avid PPGL lesion segmentation. The model has been trained using concatenated 3D CT, PET, and lesion mask images. The trained network has been validated by running inference using the PET and CT images from various clinical cohorts

Figure 1 shows the overall workflow of the AI model for automated lesion segmentation from ^68^Ga-DOTATATE PET/CT. From the nnU-Net framework, 3D full-resolution (3D-FullRes), configuration was used [10] for model development. nnU-Net was chosen because of its previously reported promising performance on medical image segmentation problems [10]. nnU-Net uses 3D U-Net architecture [9] as the backbone, with an automated pipeline, consisting of pre-processing, data augmentation, and post-processing. This method uses deep supervision to finetune the hyper-parameters automatically based on a given dataset. In this research, PET images and CT images were simultaneously used as inputs for training the model. nnU-Net used 5-fold cross-validation to train and validate models. The model was trained for 1000 epochs where each epoch consists of 250 training iterations. The Patch size was set to 128 × 128 × 128. For image normalization, nnU-Net use, z-scoring for PET, and a pre-defined scheme for CT images [10]. The learning rate was initially set to 0.01 and gradually decreased using the “poly” learning rate rule using the formula (1 − epoch/epoch_max_)^0.9^ [15]. The final checkpoint for each model was based on the epoch with the best average foreground dice similarity coefficient during the training [16, 17]. After training was completed, the inference was run on the test dataset from the PPGL cohort and also on NET and ONB cohorts using a sliding window approach with the same patch size of 128 × 128 × 128. For inference, all 5-fold cross-validation models were used as an ensemble on the test and external clinical cohorts to generate the AI-predicted tumor segmentations.

### Tumor burden calculation

Scan level tumor burden was calculated for the artificial intelligence (AI) generated lesion masks and expert annotated ground truth (GT) lesion masks from all the clinical cohorts. We calculated the total number of lesions, maximum SUV of the lesion (SUVmax), total tumor volume (TTV), and total lesion uptake (TLU), using the following equations [18], for both the AI-generated mask and the GT mask:

1 $$Overall\:{SUV}_{\text{m}\text{a}\text{x}}=\text{max}(SUV\left[mask>0\right]$$

2 $$Overall\:{SUV}_{mean}=\text{mean}(SUV\left[mask>0\right])$$

3 $$Total\:Tumor\:Volume =\sum\:\left(mask>0\right)*{\text{S}}_{x}{\text{*}\text{S}}_{y}*{\text{S}}_{z}*0.001$$

4 $$Total\:Lesion\:Uptake=SUVmean*Total\:Tumor\:Volume$$

Where mask is a binary area with lesion = 1 and background = 0; S_x_, S_y_, S_z_ is the image spacing in x, y, and z direction of the voxels of the mask in mm and multiplied by 0.001 for conversion from mm^3^ to cm^3^. TLU is a volume-based parameter that measures a tumor’s somatostatin receptor type 2 (SSR2) expression by calculating the total tumor volume multiplied by the SUVmean. As TLU is a parameter that correlates both with tumor volume and SSR2 expression of a tumor, a comparison of this parameter between expert annotation ground truth and AI-generated contours showcases the performance of the AI model on tumor burden detection. In clinical practice, TLU is observed to see the tumor burden on the patient level. This parameter is used to keep track of the patient’s overall tumor burden over time for patient treatment management, and disease follow-up.

### Statistical analysis

Two approaches were adopted to evaluate the performance of the deep learning (DL) models. In the first approach, the performance of the AI models was evaluated on a scan level. A true positive (TP) scan was defined as any AI detection in a scan with positive lesions based on GT. A scan was considered a true negative (TN) scan when AI detected no lesion in a negative GT scan. A scan was considered a false positive (FP) scan when the AI mask had a positive lesion, and the GT mask had no lesion. For the false negative (FN) scans, the AI model detected no lesion, but the GT mask had at least one positive lesion for that scan. After scans were evaluated, performance metrics such as sensitivity, specificity, overall accuracy, and the dice similarity coefficient (DSC) were calculated. The DSC was calculated by considering the overlap between expert-annotated GT lesion masks and AI-generated lesion masks [16, 17]. DSC was also reported on the scan level.

In the second approach, the performance was evaluated on the lesion level, considering all the lesions from a cohort. Here, individual lesions were identified by calculating 3D connected components (cc3d) with 26 connected neighborhoods [19]. We have calculated TP, FP, FN, sensitivity, positive predict value (PPV), median sensitivity per scan, and median FP per scan.

Further statistical analysis was performed to compare quantitative SUV metrics calculated from the AI-generated masks and GT masks both at scan and lesion levels. In the scan-level tumor burden analysis, the Bland-Altman plot [20] of difference with mean difference (MD) [21] and 95% confidence intervals (CI), median of relative mean difference (RMD), interclass correlation coefficients (ICC) [22, 23] between AI and GT masks were calculated for SUVmax, TTV, and TLU. TTV and TLU were measured in cm^3^. ICC was interpreted as superior if ICC ≥ 0.8, great: 0.61 ≤ ICC < 0.8, moderate: 0.41 ≤ ICC < 0.6, low: 0.21 ≤ ICC < 0.4, and poor: ICC ≤ 0.2 [24]. For lesion-level analysis, SUVmax, TTV, and TLU differences between TP, FN, and FP lesions were evaluated using a box-and-whisker plot, and statistical significance was evaluated based on the Clustered Wilcoxon-ranked test [25]. The statistical significance level was considered when p was less than 0.05. For statistical analysis, Python and R software were used.

A two-step failure analysis was conducted for the AI model. In the first step, failure analysis was done to evaluate the anatomical locations of TP, FP, and FN lesions. A publicly available pre-trained model on CT images called “TotalSegmentator” [26] was used to generate masks containing segmentation of major anatomical structures such as the brain, lung, mediastinum, liver, spleen, and kidney. Each lesion was characterized by its membership to a major anatomical structure and the frequency of TP, FP, and FN lesions were evaluated. In the second step, the scans with FP and FN lesions were checked by an expert radiologist to verify the nature of AI misclassifications. Tumor-to-background (TBR) ratios were calculated for the TP, FN, and FP lesions from the liver for PPGL and NET cohorts due to their high hepatic background activity in the ^68^Ga-DOTATATE PET image [27]. TBR for liver lesions was not calculated for ONB as none of the scans had liver lesions. TBR was defined as the ratio of each tumor SUVmax to individual liver SUVmean, where SUVmean of normal liver is derived from the liver organ segmentation volume of “TotalSegmentator” .

## Results

Table 1 represents the detailed information about the patients and ^68^Ga-DOTATATE-PET/CT scans for all the cohorts. As reported in Table 1, the median number of scans per patient was 3 (range: 1–9), 1 (range: 1–3), 2 (range: 1–2) and the median number of lesions per scan was 30 (range: 4-277), 18.5 (range: 3–68), 8 (range: 3–17) for PPGT, NET and ONB cohorts, respectively.

### Performance evaluation of AI models

The average validation DSC for nnUNet was 0.84 (range: 0.78–0.89). The AI-predicted lesion masks generated by the models were compared with the expert annotated GT lesion masks, to evaluate the model’s performance on the PPGL test set and external validation cohorts (NET and ONB). The scan level performance of the models is presented in Table 2. For the scan level performance, nnUNet had a DSC of 0.88, with a sensitivity of 100% on the PPGL. This indicates that the model identified all positive scans and found suspicious lesions. In supplementary table S1, the number of scans with different ranges of DSC is presented. 36 scans out of 62 total scans had DSC greater than 0.9, 17 out of 62 scans had DSC between 0.8 and 0.9, and only 2 scans had DSC between 0.4 and 0.6 for the PPGL test cohort. The lesion level performance of the model on the PPGL test set and external validation cohorts is presented in Table 3. The model achieved sensitivity of 86.60%, PPV of 89.24%, 4 (0, 34) median FP per scan, 89.48% (36.84, 100) median per scan sensitivity for the PPGL test cohort on lesion level (Table 3).

**Table 2:** Scan-level performance evaluation of the best-performing AI model (3D_FullRes) on clinical cohorts

	PPGL Test	NET	ONB
TP	62	11	7
FN	0	0	1
FP	0	0	0
Sensitivity (%)	100	100	87.5
Accuracy (%)	100	100	87.5
Dice	0.88	0.6	0.67

**Table 3:** Lesion level performance of nnUNet model on clinical cohorts

	PPGL Test	NET	ONB
TP	3457	162	45
FN	535	103	28
FP	417	57	7
Sensitivity (%)	86.60	61.13	61.64
PPV (%)	89.24	74	86.54
Median per-scan sensitivity (%)	89.48 (36.84, 100)	66.67 (29.41, 100)	64.58 (0, 85.71)
Median FP per scan	4 (0, 34)	3 (0, 16)	1 (0, 2)
DSC	0.88	0.60	0.67

For the independent NET and ONB test sets, nnUNet achieved scan-level DSC of 0.6, and 0.57, and sensitivity of 100%, and 85.71%, respectively (Table 2). Only one out of six ONB scans were identified as a false negative scan by the AI model. For the NET cohort, 2 scans had a DSC over 0.9, and two scans had a DSC less than 0.4 (supplementary table S1). For the ONB cohort, 2 scans had a DSC over 0.8, five scans had a DSC between 0.6 and 0.8, and only one scan had dice 0 (supplementary table S1). On the lesion level, the model achieved, the sensitivity of 61.13%, and 61.64% and PPV of 74%, and 86.54%, and 3 (0, 16) and 1 (0, 2) median FP per scan, 66.67% (29.41%, 100%) and 64.58%(0%, 85.71%) median per scan sensitivity were achieved in the NET, and ONB cohorts, respectively (Table 3).

### Tumor burden analysis

**Fig. 2:**
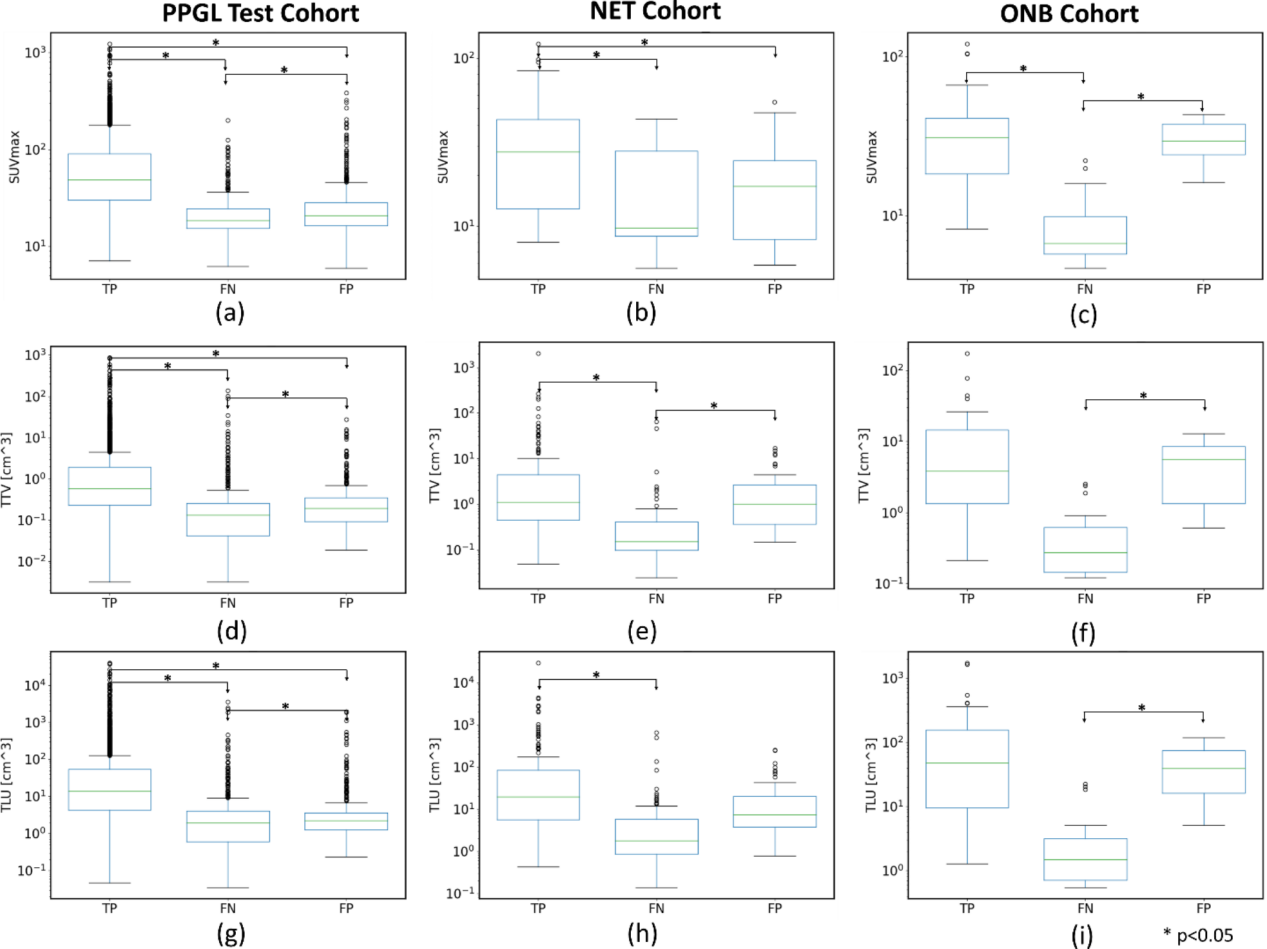
Comparison of each TP, FN, and FP lesions SUVmax (top row) for (**a**) PPGL Test, (**b**) NET (**c**) ONB cohorts, TTV [cm^3^] (middle row) for (**d**) PPGL Test, (**e**) NET, (**f**) ONB cohorts and TLU [cm^3^] (bottom row) for (**d**) PPGL Test, (**e**) NET, (**f**) ONB cohorts. Statistical significance was tested using the clustered Wilcox test and only the statistically significant differences (* *p* < 0.05) are shown with the paired line between lesion types. Without any annotations, they are not statistically significant pairwise (*p* > 0.05)

Figure 2 shows the box-and-whisker plot and Supplementary Table S2 reports the summary statistics for TP, FP, and FN lesions TTV, TLU, and SUVmax with median with 0.25 (Q1) and 0.75 (Q2) quartile values and interquartile range (IQR) for the PPGL Test, NET, and ONB cohorts. From Fig. 2, it can be observed that, for the PPGL Test, NET, and ONB cohorts, the median value of SUVmax and TTV, of FN lesions was lower than TP and FP lesions. Based on the clustered Wilcox test (Fig. 2), statistically significant differences (*p* < 0.05) were observed between different lesion types (TP, FP, FN) TTV, and SUVmax values for the PPGL and ONB cohorts. No statistically significant differences (*p* > 0.05) were found between TP, and FP tumors TTV and FN and FP tumors SUVmax and TTV values for the NET cohort. From this analysis, it can be concluded that the AI model missed lesions with lower uptake and smaller TTV in the PPGL test and ONB cohorts, as well as lesions with lower uptake in the NET cohort.

Supplementary Figure S1 shows the Bland-Altman plot of the difference between predicted and ground truth masks, SUVmax, TTV, and TLU for all the cohorts. The value of MD with 95% CI is demonstrated in Supplementary Table S3 for all the cohorts. The mean difference for TTV and TLU for all the cohorts was below zero for AI prediction masks compared to the GT masks, indicating lower tumor burden by AI due to missed lesions (Supplementary Figure S1). The mean difference for SUVmax was zero for the PPGL test and ONB cohort, an indication of great agreement in SUVmax comparison between AI and GT lesions. Four scans outside or nearest to the negative side of the 95% CI, considered outliers were observed for the PPGL test set in TTV, and TLU, indicating a high number of missed lesions by the AI-predicted mask. Among these 4 scans, 3 were scans from the same patient. Two scans were observed as outliers in the SUVmax, which fell outside of the positive side of the 95% CI, due to FP in the kidney. For the NET cohort, most of the scans had zero mean difference in TTV, SUVmax, and TLU, and only one scan was an outlier, outside the negative side of the 95% CI. AI was unable to detect 12 out of 17 lesions in the liver for this patient (supplementary figure S2). For the ONB cohort, AI was not able to detect any lesion for one scan (DSC = 0), and this case has been discussed in the failure analysis section below (supplementary figure S3).

Table 4 presents the median RMD and ICC for different tumor burden parameters, calculated between AI and GT lesion masks for different clinical cohorts. In the PPGL test cohort, the median RMD indicated a zero to almost negligible difference between the AI-predicted and the GT masks for all tumor burden parameters. Very negligible differences between the AI and GT masks were observed for the NET and ONB cohorts for all the tumor burden parameters (Table 4). Table 4 shows the ICC between AI-generated masks and expert-annotated GT masks, revealing that SUVmax had excellent ICC values (0.99-1) for the PPGL test, NET, and ONB cohorts. For the PPGL test and ONB cohorts, superior ICC was observed for TTV and TLU (range 0.8–0.98), whereas for the NET cohort, moderate ICC was observed for these parameters (range 0.66–0.76).

**Table 4:** Median RMD and ICC of different tumor burden parameters between AI and GT lesion masks for different clinical cohorts

	PPGL Test		NET		ONB	
	Median RMD	ICC	Median RMD	ICC	Median RMD	ICC
SUVmax	0.00 (-0.30, 2.57)	0.992	0.00 (0.00, 2.71)	1	0.00 (-1, 0)	0.998
SUVmean	0.04 (-0.08, 1.75)	0.967	0.07 (-0.27, 0.69)	0.903	0.23 (-1, 0.32 )	0.836
TTV	-0.10 (-0.69, 0.26)	0.913	-0.20 (-0.99, 1.90)	0.663	-0.33 (-1, -0.21 )	0.792
TLU	-0.03 (-0.97, 38.94)	0.981	-0.09 (-0.98, 1.12)	0.759	-0.17 (-1, -0.06 )	0.953

### Failure analysis

**Fig. 3:**
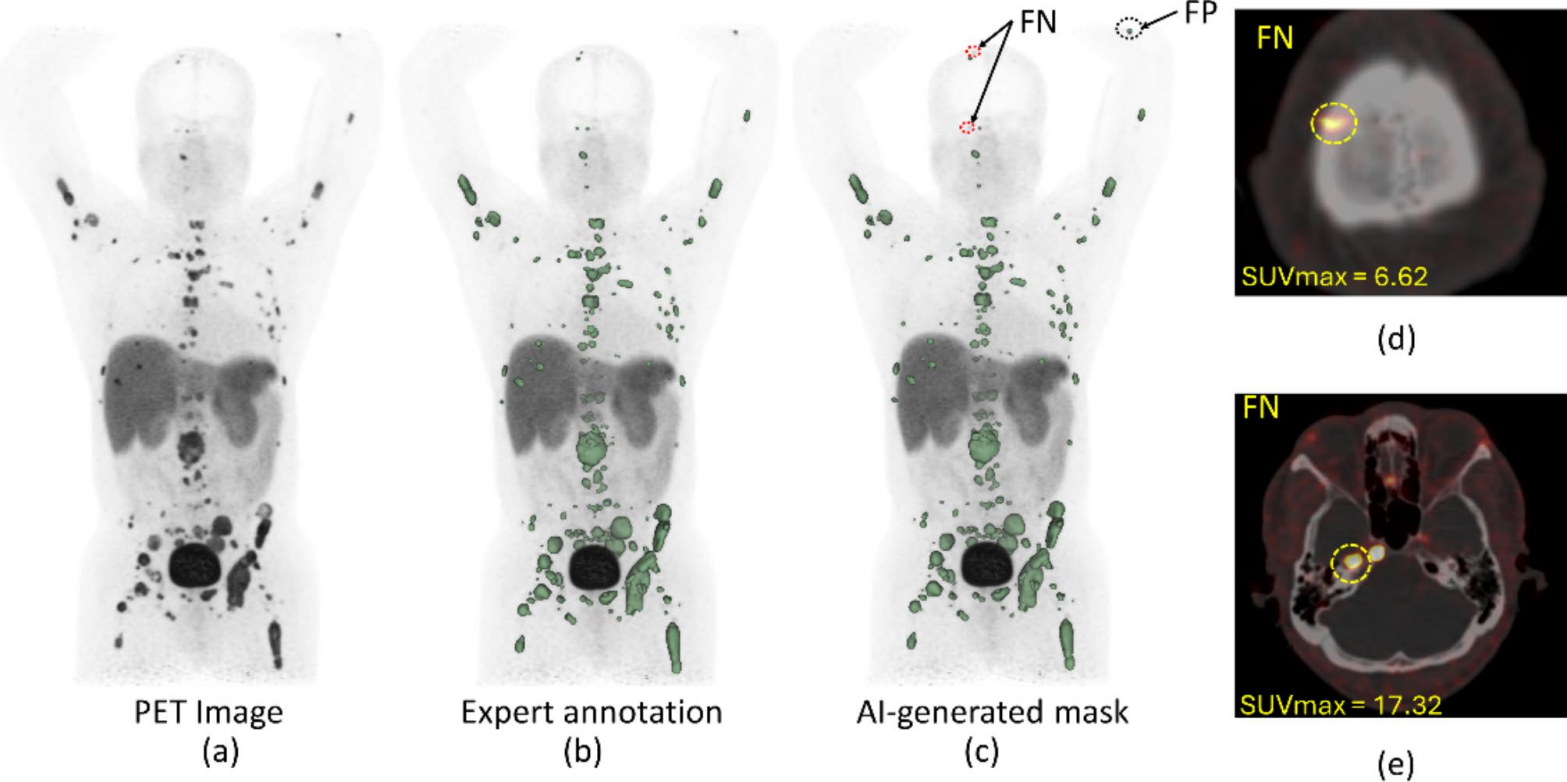
(**a**) ^68^GA-DOTATATE PET maximum intensity projection (MIP) image of a patient from the PPGL cohort, (**b**) lesion annotations by experts (**c**) lesion annotations by artificial intelligence (AI) model with the marking of one false positive (FP) (black dotted circle) and two false negative (FN) lesions) (yellow dotted circle) (DSC = 0.96). (**d**)-(**e**) Axial PET/CT images, showcasing two FN lesions (SUVmax of 6.62, 17.32) (marked in a yellow dotted circle) by AI

**Fig. 4:**
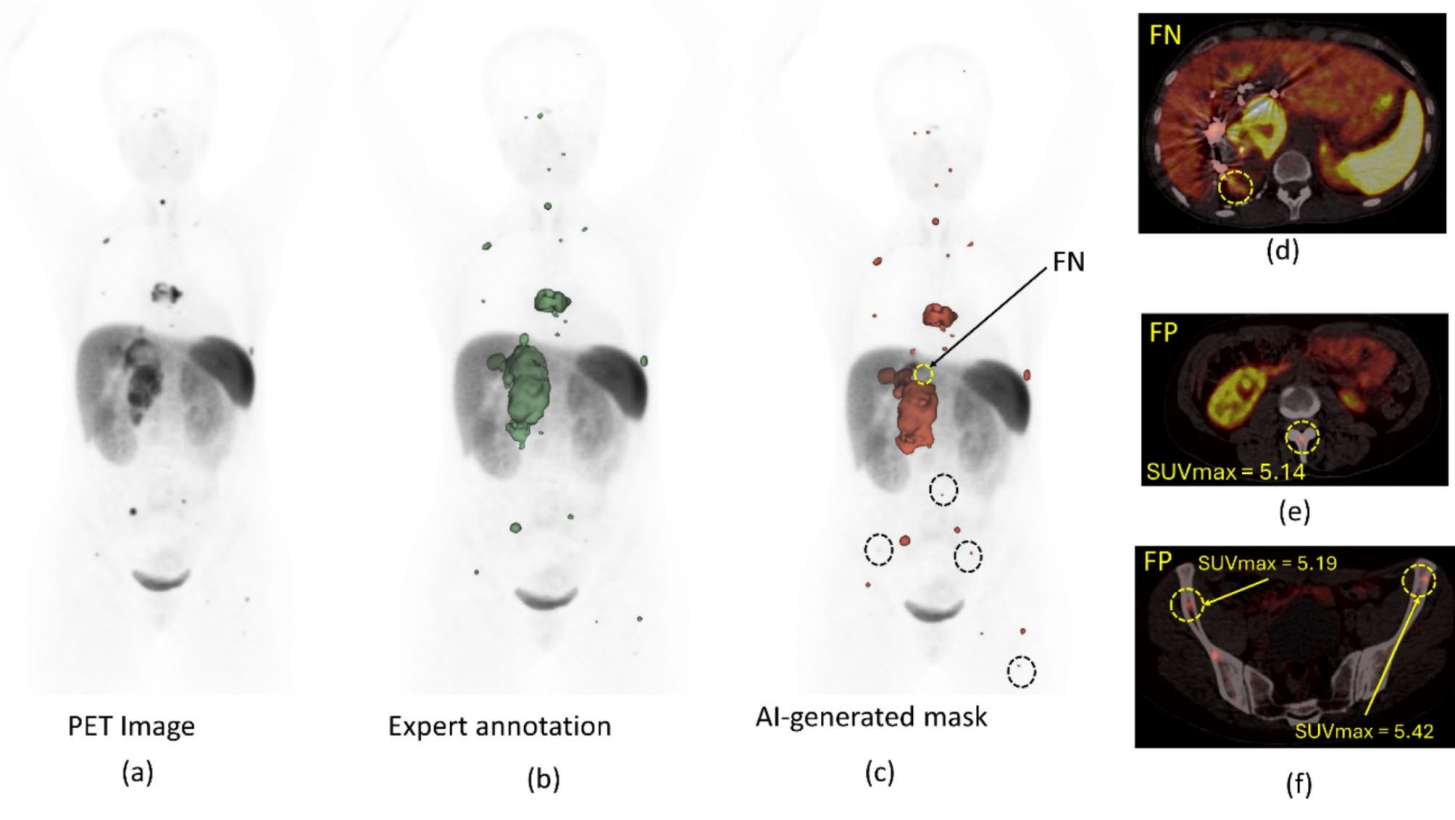
(**a**) ^68^GA-DOTATATE PET maximum intensity projection (MIP) image of a patient from the PPGL cohort, (**b**) lesion annotations by experts (**c**) AI predicted lesion annotation (DSC = 0.86)) with one false negative (FP) (yellow dotted circle) and multiple very small false positive lesions (black dotted circle). (**d**) Axial PET/CT images, showcasing the FN lesion by AI. (**e**)-(**f**) Axial PET/CT images, showcasing multiple FP lesions by AI marked in a yellow dotted circle. AI identified bone lesions with SUVmax in the range 5.14 to 5.42, which have been missed in the expert annotation due to using a semi-automated threshold (SUV ≥ 6) to create the annotations

**Fig. 5:**
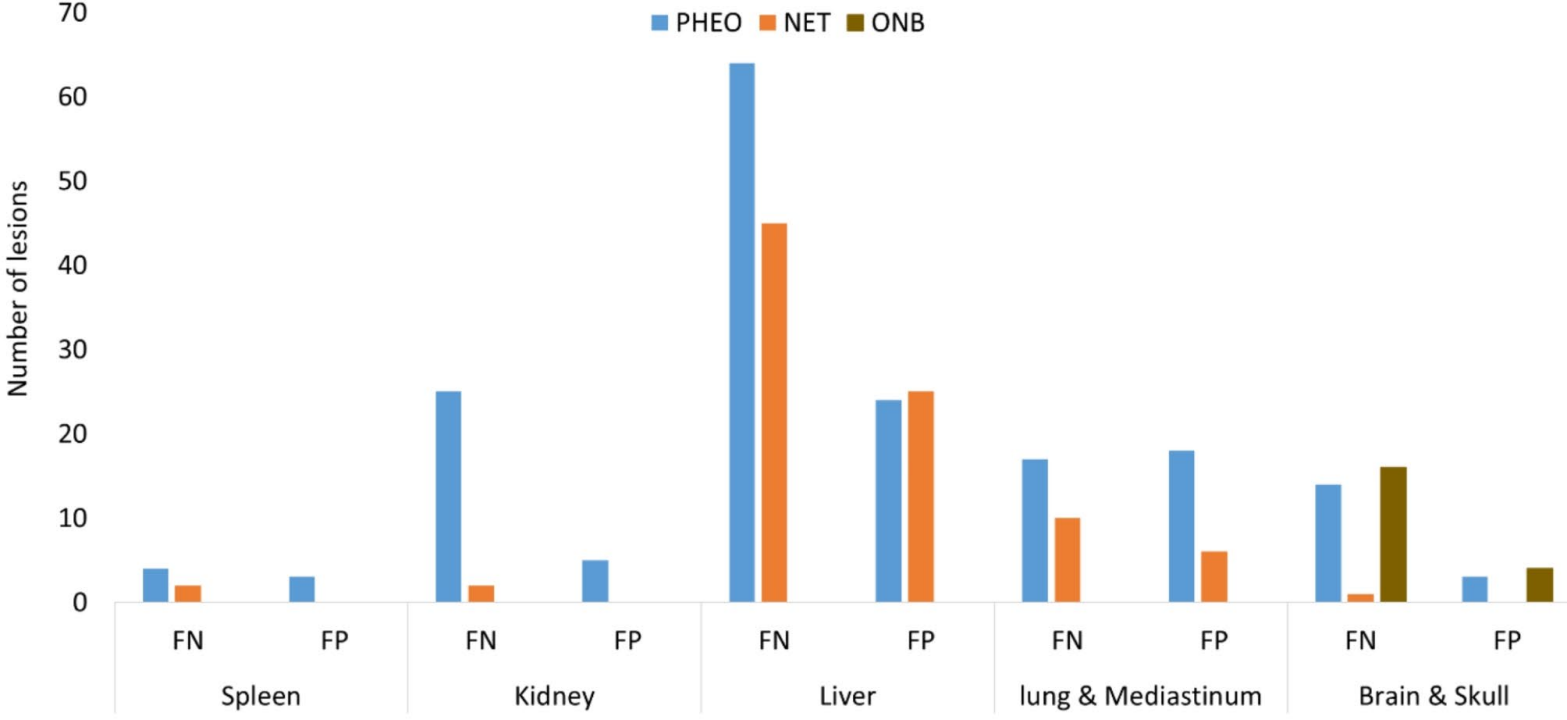
Histogram representation of the frequency of different types of lesions in different anatomical structures for all the cohorts

In all cohorts, FP, and FN lesions were observed and these were further interrogated (Figs. 3 and 4). Figure 5 shows a histogram containing the frequency of TP, FN, and FP lesions by anatomical site. For both the PPGL test and NET cohort, the highest number of FN and FP lesions were in the liver (Fig. 5). For the ONB cohort, most of the FN lesions were in the brain & skull region. Supplementary Figure S2 showcases the TBR for the TP, FN, and FP liver lesions. The median value of FN liver lesions TBR is significantly smaller than TP liver lesions TBR, which indicates why the FN lesions are missed by the AI model. Statistically significant differences were observed in TBR for TP and FN lesions for PPGL and NET cohort. Additional case samples of our failure analysis are presented in Figures S3 and S4 in the supplementary material. In supplementary figure S3, an example NET patient was showcased (DSC = 0.37) where a large liver mass was missed by AI. However, upon the investigation of the overlay PET/CT image for this scan, it was observed that an area annotated by the expert with a low signal-to-background ratio relative to the physiological liver uptake. (supplementary figure S3(e)). In supplementary figure S4, another example is shown for an ONB patient. AI was able to detect almost all the lesions (DSC = 0.87) from the baseline scan (supplementary figure S4 (a)). However, AI was not able to detect any lesions in this patient after treatment. Supplementary figure S4 (b) showed that after treatment all the lesions almost recovered or shrank with very small residual areas with a total tumor volume of 1.9 cm^3^ and overlay of PET/CT images supplementary figure S4 (c) showed a very small uptake for these residual areas which was missed by AI.

In Supplementary Table S4, the Median SUVmax value of TP, FN, and FP lesions in different anatomical locations were reported. No significant difference was observed in the median SUVmax values between TP, FN, and FP lesions in those anatomical regions for the PPGL and NET cohort, however, for the ONB cohort, low uptake lesions were missed (median SUVmax 6.41 (4.84, 22.19)).

## Discussion

PPGL is a relatively rare cancer for which there are few specific imaging methods. The use of ^68^Ga-DOTATATE for PET imaging is increasing because of its advantage in planning targeted peptide receptor radionuclide therapy (PRRT), mostly used for NET tumors such as PPGL [2]. With the significant breakthrough in AI technologies, manual and laborious lesion annotation processes can be automated to save time, reduce the work burden of nuclear imaging physicians, and improve consistency. In this study, we have built an AI tool based on 3D full-resolution nnUNet configuration, for automatic PPGL lesion segmentation, using a clinical cohort who were undergoing ^177^LU-DOTATATE PRRT. The model achieved a lesion level DSC of 0.88 and a sensitivity of 86% on the PPGL test cohort.

Prior reports using AI for NET segmentations have been published. Wehrend et al. [28] developed a hepatic lesion segmentation model based on ^68^Ga-DOTATATE PET/CT images from 125 patients using 2D U-Net architecture and reported a sensitivity of 74%. Carlsen et al. [7] developed a 3D FullRes nnUNet-based lesion segmentation model for gastroenteropancreatic and lung neuroendocrine neoplasms (NENs) from ^64^Cu-DOTATATE PET/CT images. Their model achieved a lesion level DSC of 0.85 and a sensitivity of 0.84. Other two studies used radiomics to extract information from ^68^Ga-DOTANOC PET/CT (an agent similar to ^68^Ga-DOTATOC) for predicting tumor grading of pancreatic NET (PNET) with a sensitivity of 88% [1] and ^68^Ga-DOTATOC PET images for identification of lymph node metastases in patients with PNET with a sensitivity of 77% [22]. PPGL differs in imaging properties from NET even though it is detected with the same PET agents. In comparison with [23], and [24], our model had better sensitivity and DSC. Our AI model was further tested in 2 different clinical datasets which included 2 relatively rare cancer types and achieved a sensitivity of 61.13% and 61.64% and DSC of 0.6, and 0.57, for NET and ONB cohorts, respectively. It is worth noting that the model was trained on a PPGL cohort but also performed well in other types of NETs (NET and ONB cohorts), which indicates the generalizability of our AI model. Overall, despite this domain shift in the test population related to differences in types of NET tumors, the AI model performed well for PPGL and moderately for NET and OBN cohorts.

In the PPGL test cohort, 58% of scans had DSC over 0.9, 27% of scans had DSC between 0.8 and 0.9, and only two scans had DSC less than 0.6 but over 0.4 DSC. These results showcase that, much less effort is needed from a nuclear medicine physician for quantifications of the PPGL lesions. To further evaluate the performance of our AI model, detailed tumor burden and failure analyses were conducted, demonstrating negligible underestimation of SUVmax, TTV, and TLU for all the cohorts. It was also observed that within all the cohorts, FN and FP lesions predominantly occurred in smaller-volume lesions. Failure analysis based on the locations of FP and FN lesions in major anatomical structures such as the brain, lung, mediastinum, liver, spleen, and kidney demonstrated the highest number of FN and FP lesions in the liver region for all cohorts. One of the biggest drawbacks of the ^68^Ga-DOTATATE PET modality is high physiologic/normal hepatic background and generalized image noise which makes the detection of smaller liver lesions for our AI model [29], as special image intensity and background noise normalization techniques are needed as preprocessing steps to overcome this challenge. However, such normalizations might affect the model’s overall performance for the other normal physiological uptake regions as it might lose information due to this intense preprocessing for the liver. The tumor burden parameters for the PPGL test and ONB cohorts had ICC values ranging from 0.8 to 0.99. For NET patients, ICC values ranged (0.66-0.76) for TTV and TLU however, superior ICC was observed for SUVmax values.

^68^Ga-DOTATATE-PET/CT has the benefit of high sensitivity for detecting SSTR2 tumors and thereby selecting candidates with a strong likelihood of responding to PRRT [2, 30]. AI-assisted automated lesion segmentation tools in PPGL patients will efficiently help to identify suitable tumors for targeted PRRT and track total tumor burden over time, expediting treatment planning. It will also help with faster treatment decision-making in selecting which tumors can be operable with the better accurate staging [2, 30].

This study showed that an AI-based lesion segmentation model can be a reliable solution to reduce the workload of nuclear medicine and oncology experts from the tedious manual lesion segmentation process and tumor burden analysis. This approach can be expanded to other cancer types with PET tracers beyond FDG, such as prostate-specific membrane antigen (PSMA), fibroblast activation protein inhibitor (FAPI), Fluoroestradiol (FES), Fluorothymidine (FLT), and many others.

Our study has some important limitations. As discussed earlier, the ground truth used in PPGL, NET, and ONB cohorts was based on semi-automated, threshold-based techniques. While considerable review and manual effort were made to reduce non-disease entities from being included in the ground truth, there remains a possibility that areas of true positive disease that were below the SUV threshold were not included in the ground truth. Future evaluation of the agreement between physicians and AI-detected regions is warranted. Secondly, the model failed to detect liver lesions for most of the cohorts. Having a cascaded model, specifically trained on the liver and lung lesions, with image pre-processing steps including “tumor-to-background ratio (TBR)” can be a potential solution to overcome this limitation. Finally, because of the rarity of the condition, our study sample is relatively small, and it is clear that our AI model needs further testing using external datasets. In the future, we will plan to work on different pre-processing steps i.e. systematically adding noise to the data, for DOTATATE-PET/CT scans that could be harnessed to best help the AI tool perform optimally. Furthermore, synthetic digital lesions could be added to PET scan data for a healthy volunteer to precisely tune characteristics to explore the influence of specific pre-processing strategies.

## Conclusions

In conclusion, this AI model developed for automated segmentation of PPGL lesions from ^68^Ga-DOTATATE-PET/CT images delivers reliable performance metrics with an acceptable range and category of FP lesions. AI-generated segmentation in PPGL will enhance patient selection for PRRT, provide consistent serial tumor monitoring as well as improve workflow efficiency. Additionally, AI-generated segmentation may significantly reduce the time physicians spend on manual lesion annotation, which is particularly beneficial in the post-treatment follow up with ^68^Ga-DOTATATE-PET/CT. This is important progress with clinical benefit in the evaluation of PPGL tumors on 68Ga-DOTATATE-PET/CT and speeding the process by which patients begin therapy and more accurately assessing response to that therapy.

## Electronic supplementary material

Below is the link to the electronic supplementary material.

Supplementary Material 1

## Data Availability

The datasets used in this study are not yet publicly available as these are from ongoing clinical trials.

## Code availability

The model weights have been published with instructions in the following GitHub repository: https://github.com/HFahmida/PPGL_AI.gi.
