# Supplementary material for: An automated pheochromocytoma and paraganglioma lesion segmentation AI-model at whole-body 68Ga- DOTATATE PET/CT
Source: EJNMMI Res. 2024 Nov 5;14:103. doi: 10.1186/s13550-024-01168-5 (PMC11538206; doi:10.1186/s13550-024-01168-5)
Supplement: Supplementary file 1 — Supplementary Material 1 [file 13550_2024_1168_MOESM1_ESM.docx]

**An Automated Pheochromocytoma and Paraganglioma lesion segmentation AI-model at whole-body ^68^Ga- DOTATATE PET/CT**

Fahmida Haque^1^, Jorge A. Carrasquillo^2^, Evrim B. Turkbey^3^, Esther Mena^2^, Liza Lindenberg^2^, Philip C. Eclarinal^2^, Naris Nilubol^4^, Peter L. Choyke^2^, Charalampos S. Floudas^5^, Frank I. Lin^2^, Baris Turkbey^1,2^ , Stephanie A. Harmon^1^

^1^Artificial Intelligence Resource, National Cancer Institute, National Institutes of Health, Bethesda, MD, 20814, USA

^2^Molecular Imaging Branch, National Cancer Institute, National Institutes of Health, Bethesda, MD, 20814, USA

^3^Radiology and Imaging Sciences, Clinical Center, National Institutes of Health, Bethesda, MD, 20892, USA

^4^Surgical Oncology Program, National Cancer Institute, National Institutes of Health, Bethesda, MD, 20892, USA

^5^Center for Immuno-Oncology, National Cancer Institute, National Institutes of Health, Bethesda, MD, 20892, USA

**Table S1:** The number of scans in different dice scores ranges from all the cohorts.

|  | number of scans/total number of scans | | |
| --- | --- | --- | --- |
| Dice | **PPGL Test** | **NET** | **ONB** |
| dice ≥ 0.9 | 36/62 | 2/11 | - |
| dice ≥ 0.8 | 17/62 | - | 2/8 |
| 0.6≤ dice<0.8 | 7/62 | 4/11 | 5/8 |
| 0.4≤dice<0.6 | 2/62 | 3/11 | - |
| dice<0.4 | - | 2/11 | 1/8 |

**Table S2:** Summary Statistics table for TP, FP, and FN lesions TTV, TLU, and SUVmax

| Tumor burden parameters | Cohort | Lesion type | Lesion count | mean | SD | median | Q1 | Q3 | IQR |
| --- | --- | --- | --- | --- | --- | --- | --- | --- | --- |
| SUVmax | PHEO | FN | 535 | 23.02 | 16.52 | 18.50 | 15.41 | 24.38 | 8.97 |
|  |  | FP | 417 | 31.84 | 39.93 | 20.68 | 16.28 | 28.05 | 11.78 |
|  |  | TP | 3457 | 76.35 | 85.57 | 48.52 | 29.97 | 89.94 | 59.97 |
|  | NET | FN | 103 | 16.34 | 10.98 | 9.78 | 8.74 | 27.97 | 19.23 |
|  |  | FP | 57 | 17.57 | 10.76 | 17.25 | 8.34 | 24.58 | 16.25 |
|  |  | TP | 162 | 30.19 | 20.16 | 27.65 | 12.72 | 43.07 | 30.36 |
|  | ONB | FN | 28 | 8.41 | 4.41 | 6.68 | 5.74 | 9.91 | 4.16 |
|  |  | FP | 7 | 30.30 | 9.91 | 29.50 | 24.10 | 37.60 | 13.50 |
|  |  | TP | 45 | 35.20 | 24.90 | 30.90 | 18.30 | 41.10 | 22.80 |
| TTV | PHEO | FN | 535 | 1.24 | 8.69 | 0.13 | 0.04 | 0.26 | 0.22 |
|  |  | FP | 417 | 0.66 | 2.20 | 0.19 | 0.09 | 0.35 | 0.26 |
|  |  | TP | 3457 | 5.55 | 38.01 | 0.58 | 0.23 | 1.94 | 1.71 |
|  | NET | FN | 103 | 1.43 | 7.77 | 0.15 | 0.10 | 0.41 | 0.31 |
|  |  | FP | 57 | 2.40 | 3.73 | 1.00 | 0.36 | 2.64 | 2.28 |
|  |  | TP | 162 | 23.00 | 164.01 | 1.11 | 0.44 | 4.41 | 3.96 |
|  | ONB | FN | 28 | 0.53 | 0.66 | 0.27 | 0.14 | 0.62 | 0.47 |
|  |  | FP | 7 | 5.54 | 4.71 | 5.62 | 1.34 | 8.50 | 7.17 |
|  |  | TP | 45 | 13.30 | 28.50 | 3.83 | 1.34 | 14.60 | 13.20 |
| TLU | PHEO | FN | 535 | 29.83 | 226.99 | 1.97 | 0.60 | 4.06 | 3.46 |
|  |  | FP | 417 | 23.82 | 149.59 | 2.24 | 1.28 | 3.63 | 2.35 |
|  |  | TP | 3457 | 222.09 | 1583.15 | 13.84 | 4.34 | 53.88 | 49.54 |
|  | NET | FN | 103 | 307.45 | 1430.59 | 151.87 | 97.80 | 405.25 | 7766.25 |
|  |  | FP | 57 | 16.10 | 26.14 | 7.34 | 3.74 | 19.84 | 49.56 |
|  |  | TP | 162 | 77.76 | 394.91 | 19.39 | 5.57 | 83.33 | 2369.84 |
|  | ONB | FN | 28 | 3.72 | 6.00 | 1.48 | 0.71 | 3.14 | 2.42 |
|  |  | FP | 7 | 48.80 | 41.30 | 39.00 | 16.10 | 73.70 | 57.70 |
|  |  | TP | 45 | 176.00 | 355.00 | 47.40 | 9.58 | 156.00 | 146.00 |

**Table S3:** Mean difference with 95% confidence interval in different tumor burden parameters by AI and GT masks on patient level.

|  | **PPGL Test** | **NET** | **ONB** |
| --- | --- | --- | --- |
| **SUVmax** | 5.15  (-60.02, 70.32) | 6.05  (-34.12, 46.21) | -0.82  (-5.38, 3.73) |
| **Total tumor volume** | -49.21  (-282.92, 184.50) | -236.94  (-1378.63, 904.76) | -24.56  (-60.47, 11.36) |
| **Total lesion uptake** | -1012.24  (-5975.14, 3950.67) | -3419.71  (-19663.21, 12823.79) | -147.57  (-391.37, 96.24) |

**Table S4:** Median SUVmax of the lesions from different anatomical positions and anatomical positions with the highest number of lesions for different lesion types of the cohorts.

| Cohort | Lesion Type | Median SUVmax  (Min, Max) | Median SUVmax of the lesions from the anatomical position with the highest number of lesions | |
| --- | --- | --- | --- | --- |
|  |  |  | Anatomical Position | Median SUVmax  (Min, Max) |
| PPGL | TP | 63.08  (7.13, 611.19) | Lung & Mediastinum | 58.33  (11.23, 611.19) |
|  | FN | 24.45  (7.67, 125.44) | Liver | 20.06  (8.08, 93.51) |
|  | FP | 46.52  (5.96, 384.83) | Liver | 50.69  (5.96, 323.48) |
| NET | TP | 36.54  (8.19, 121.43) | Liver | 43.13  (14.19, 121.43) |
|  | FN | 13.43  (5.63, 43.47) | Liver | 29.23  (7.16, 43.47) |
|  | FP | 24.50  (5.86, 34.87) | Liver | 24.58  (10.86, 34.87) |
| ONB | TP | 25.86  (8.24, 104.37) | Brain & Skull | 25.86  (8.24, 104.37) |
|  | FN | 6.41  (4.84, 22.19) | Brain & Skull | 6.41  (4.84, 22.19) |
|  | FP | 31.98  (27.05, 40.77) | Brain & Skull | 31.98  (27.05, 40.77) |
|  | | | | |


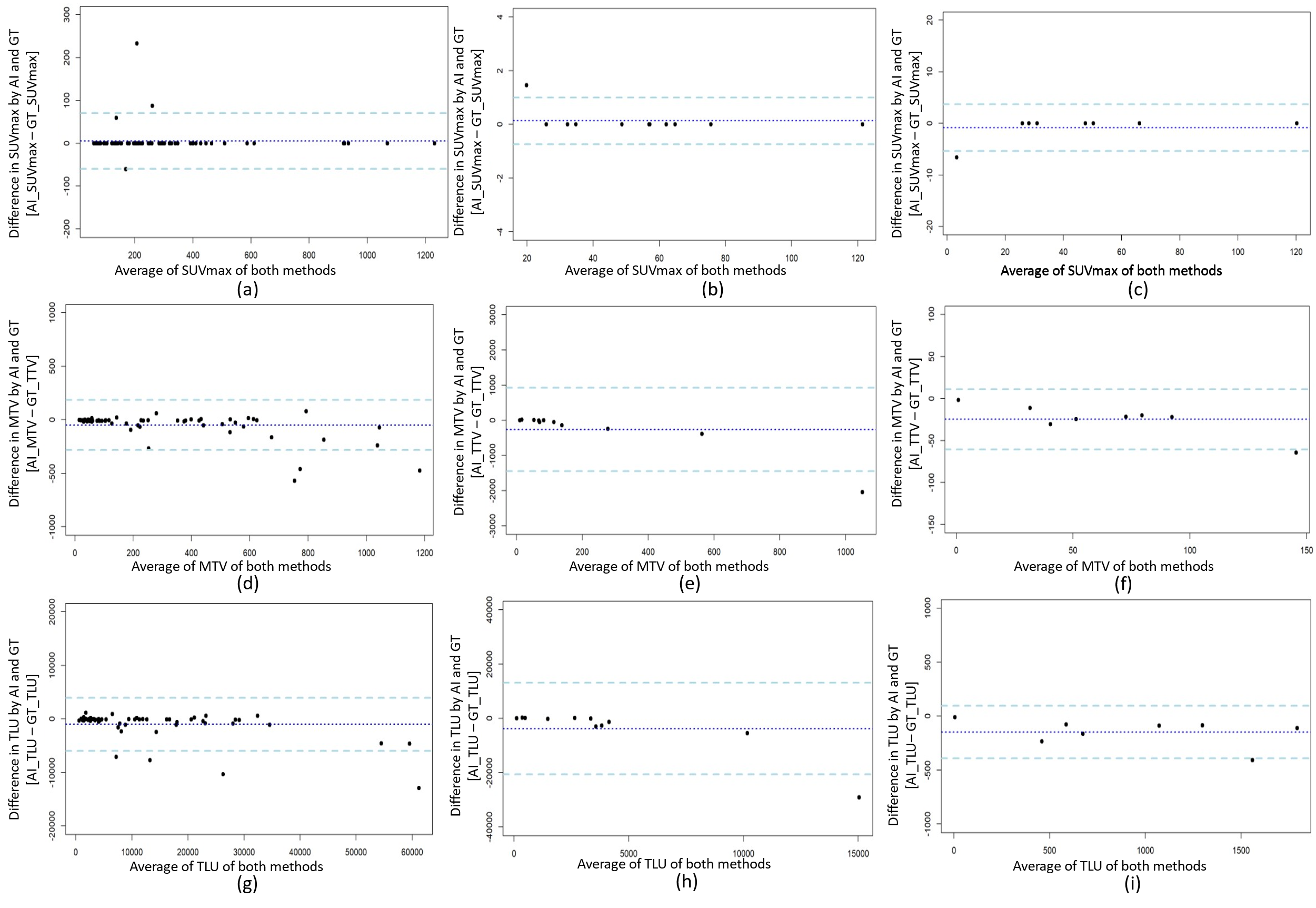


**Figure S1:** Bland-Altman plot of the difference for SUVmax (top row) for (a) PPGL test, (b) NET, (c) ONB cohorts, total tumor volume (TTV) (middle row), (d) PPGL test, (e) NET, (f) ONB cohorts and Total lesion uptake (TLU) (bottom row) (g) PPGL test, (h) NET, (i) ONB cohorts between AI generated and GT expert annotated lesion masks.


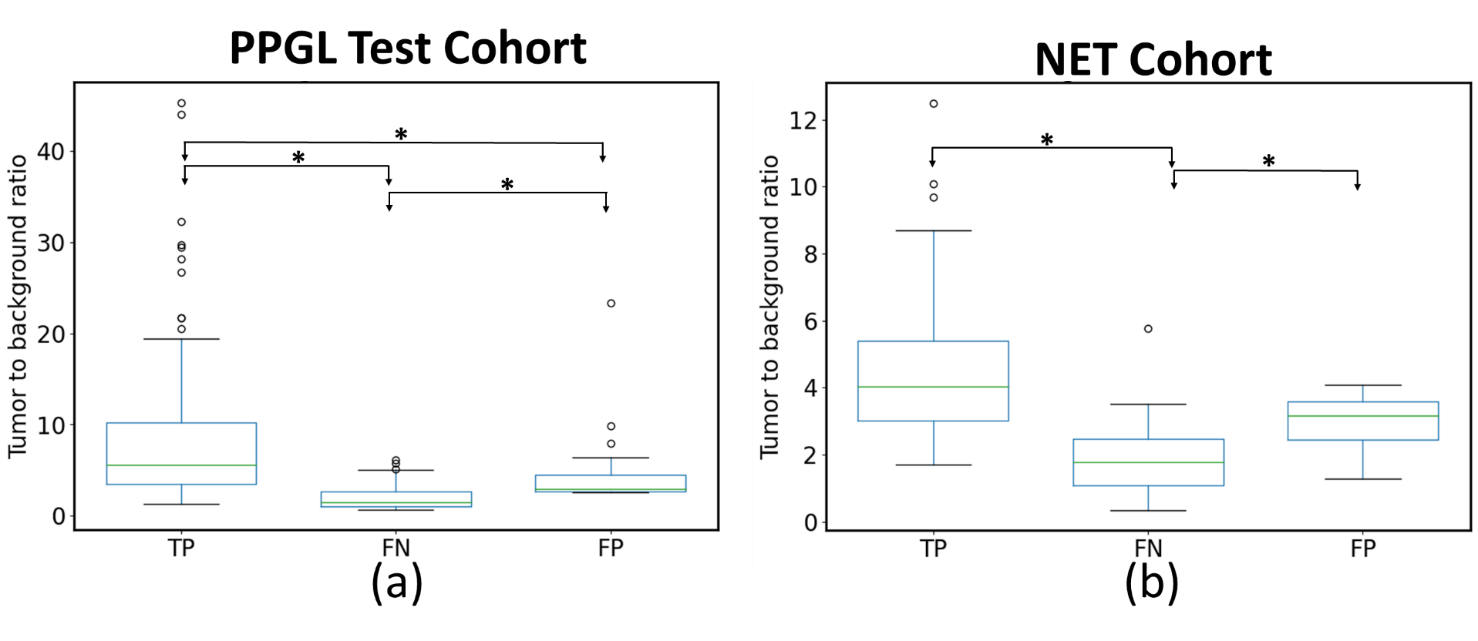


**Figure S2:** Box-and-Whisker plot of each TP, FN, and FP liver lesions tumor-to-background ratio (TBR) (a) PPGL Test, (b) NET cohorts. Statistical significance was tested using the clustered Wilcox test and only the statistically significant differences (* p<0.05) are shown with the paired line between lesion types. Without any annotations, they are not statistically significant pairwise (p>0.05).


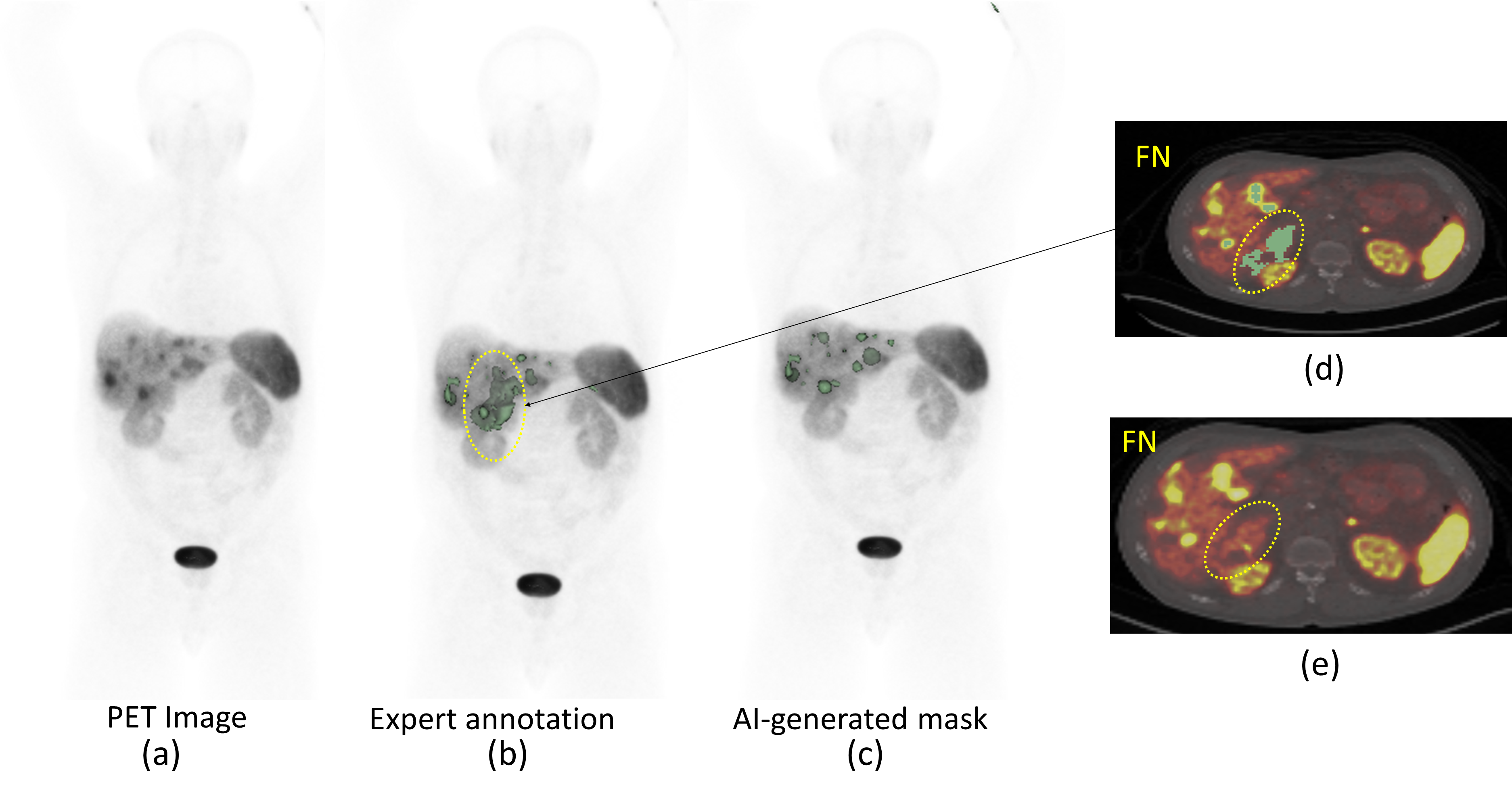


**Figure S3:** NET cohort example. **(a)** Maximum Intensity Projection (MIP) image, non-annotated. **(b) MIP with** Expert annotations. Lesions not detected by AI model, (False Negative (FN)) are in yellow dotted circle.. (c) MIP with lesion annotation by AI model with dice 0.37 (Consider adding circle to demonstrate FN miss.) **(d)** Axial PET/CT images with FN lesion in the liver region (missed lesion by AI) marked by an expert in the dotted yellow circle. **(e)**Axial PET/CT images of the same FN lesion (as (d)) marked by an expert in the dotted yellow circle. This lesion had a low tumor uptake-to-background ratio relative to the physiological liver uptake.


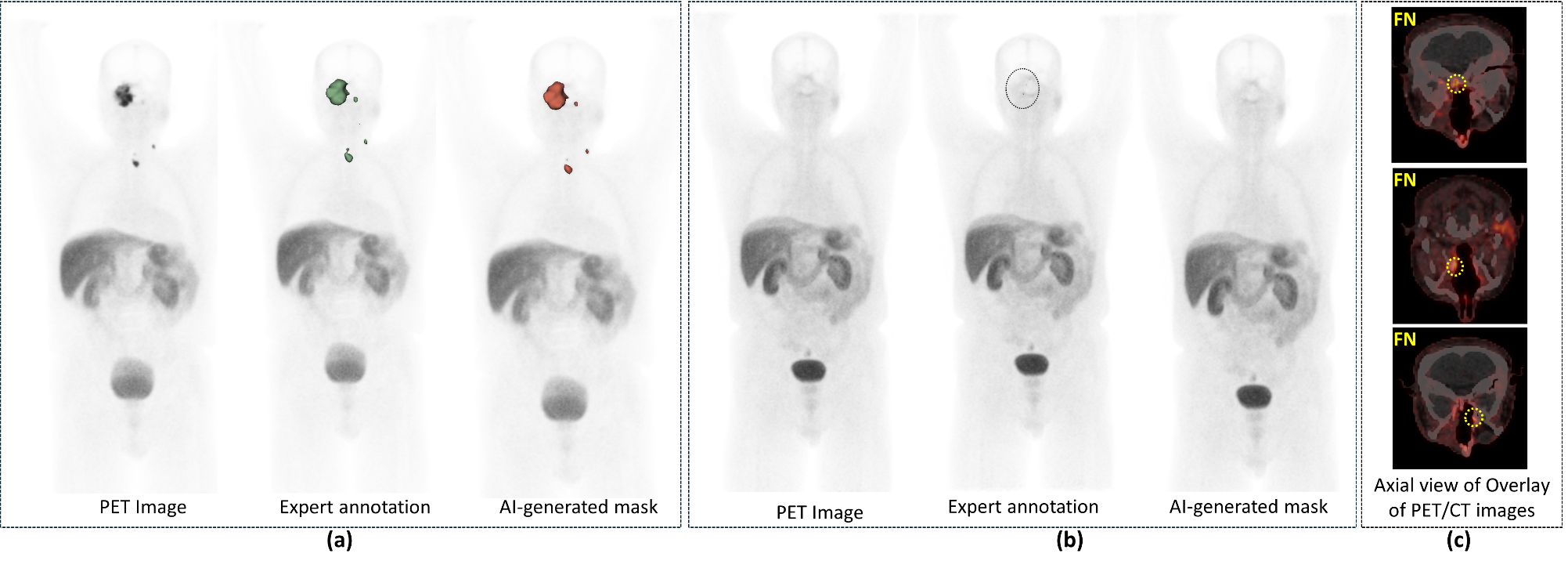


**Figure S4:** Example from ONB cohort (a) baseline maximum intensity projection (MIP) PET images without and with expert annotations (tumor annotation in green) and AI-generated mask (tumor annotation in red). The scan achieved a DSC of 0.87. (b) After treatment MIP PET images of the same patient where the expert annotation (tumor annotation in green, marked in a black dotted circle) show drastic shrinkage of the tumors, with some residual tumor, total volume = 1.91 cm^3^. AI was not able to detect any residual lesion areas (DSC = 0). (c) Axial PET/CT images, showcasing multiple false negative (FN) lesions by AI (marked in a yellow dotted circle).
